# The ascent of man(made oxidoreductases)

**DOI:** 10.1016/j.sbi.2018.04.008

**Published:** 2018-08

**Authors:** Katie J Grayson, JL Ross Anderson

**Affiliations:** 1 School of Biochemistry, Biomedical Sciences Building, University of Bristol, BS8 1TD, UK; 2 BrisSynBio Synthetic Biology Research Centre, Life Sciences Building, University of Bristol, Tyndall Avenue, Bristol BS8 1TQ, UK

## Abstract

Though established 40 years ago, the field of *de novo* protein design has recently come of age, with new designs exhibiting an unprecedented level of sophistication in structure and function. With respect to catalysis, *de novo* enzymes promise to revolutionise the industrial production of useful chemicals and materials, while providing new biomolecules as plug-and-play components in the metabolic pathways of living cells. To this end, there are now *de novo* metalloenzymes that are assembled *in vivo*, including the recently reported C45 maquette, which can catalyse a variety of substrate oxidations with efficiencies rivalling those of closely related natural enzymes. Here we explore the successful design of this *de novo* enzyme, which was designed to minimise the undesirable complexity of natural proteins using a minimalistic bottom-up approach.

## Highlights

• Here we highlight our recent advances in *de novo* enzyme design.
• Heme C-binding maquettes with minimal complexity serve as promiscuous and efficient enzymes.
• Well-defined substrate binding sites are possibly not required for efficient oxidoreductase catalysis.

**Current Opinion in Structural Biology** 2018, **51**:149–155

This review comes from a themed issue on **Engineering & design**

Edited by **Giovanna Ghirlanda** and **Ivan Korendovych**

For a complete overview see the Issue and the Editorial

Available online 10th May 2018

**https://doi.org/10.1016/j.sbi.2018.04.008**

0959-440X/© 2018 The Authors. Published by Elsevier Ltd. This is an open access article under the CC BY license (http://creativecommons.org/licenses/by/4.0/).

## The design of novel proteins

*De novo* protein design has its roots in the 1970s with the design of functional peptides that aimed to understand the rules governing the relationship of amino acid sequence with higher order structure and function [1, 2, 3]. Since then, the field has flourished with advances in technology, protein structure prediction and recombinant protein expression [4^••^], with designs that mimic [5, 6], supersede [7] or perform chemistry not seen in nature [8]. Through this we may further our understanding of how natural proteins operate [9], or augment the functional possibilities currently available to us from nature's repertoire of proteins. The simplicity of most *de novo* proteins is an advantage over the often complex and intricate characteristics and interactions of natural proteins; millennia of natural selection have imprinted and consolidated this complexity on natural proteins, with many individual amino acids becoming irreversibly dependent on each other [10, 11]. Therefore, it is not always straightforward to replicate function in a *de novo* designed protein by importing natural sequences. In this review we discuss a bottom-up approach to *de novo* protein design, based on bundles of four alpha-helices, in which the complexity of natural proteins is avoided.

The first *de novo* four-helix bundle proteins were designed in the late 1980s by DeGrado in which repeated amino acid heptads form a structure with each individual amino acid having a well-defined role [12]. Heptads of amino acid residues with high helical forming propensities form two turns of an alpha helix, helix length can be tailored by building up a series of heptad repeats, and linked with loops containing residues with low helix-forming propensities. Protein folding is driven by the exclusion of water from the protein core through the patterning of polar and nonpolar residues [12]. These simple principles have formed the basis of many *de novo* protein designs, although there are designs that are made up of beta-strand elements [13, 14, 15].

Novel protein scaffolds may be designed rationally to achieve a particular function, and/or use directed evolution to evolve towards or refine the desired activity. Developments in high throughput techniques have facilitated the construction and screening of large protein libraries [16, 17], while the use of computational design has increasingly allowed us to design scaffolds whose experimentally determined structure remains faithful to that of the intended design [15]. While many *de novo* proteins were designed with a specific function in mind [6], other functionalities were more serendipitous [18]. Many designs take inspiration from natural structures [19], or incorporate natural sequences [20].

In this review we discuss a strategy for the successful design of *de novo* enzymes, and in particular we discuss our use of the maquette approach [21^•^], in which we design an evolutionary naïve, robust structure built from the minimum number of amino acids possible, and use a cofactor to imprint function [22^••^]. In this review we challenge some design principles surmised from natural proteins: complexity, specificity, and a defined structure, and examine whether they are strictly necessary for an effective *de novo*-designed biological catalyst.

## The maquette approach to protein design

Maquettes are *de novo*-designed self-assembling peptide scaffolds pioneered by Dutton and colleagues. They are designed bottom-up without mimicking natural sequences with the intention of minimising the complexity present in naturally evolved proteins, and are subject to iterative rounds of design with significant engineering freedom [21•, 23, 24]. Maquette functions to date are diverse, recent examples include light harvesting [25] and subsequent energy transfer [26], oxygen binding [27], oxidation and oxidative dehalogenation catalysis [22^••^], amphiphilic maquettes for transmembrane electron transfer [28], and magnetic field sensing [29].

Function is conferred onto a maquette scaffold through the incorporation of cofactor molecules, and many maquette designs contain heme. A large amount of natural proteins contain heme, exhibiting an exceptionally diverse range of functions including electron transfer, catalysis, sensing, and transport [30], and in fact heme may have been utilised by early enzymes as a way to incorporate activity [18, 31]. Many heme-containing proteins are alpha helical structures, including simple 4-helix bundles [18, 30], the scaffold used in most maquette designs. Heme has therefore proven a useful cofactor to incorporate into artificial proteins, particularly due to the ease by which it can be incorporated: heme B can be ligated through two histidine residues on the interior faces of neighbouring helices, and multiple hemes can be bound within a monomeric scaffold [32, 33]. The first maquette design was based on the bis-histidine heme binding sites in the respiratory *bc*_1_ complex [19], with the heme-ligating residues located along the hydrophobic helix interfaces.

**Figure 1:**
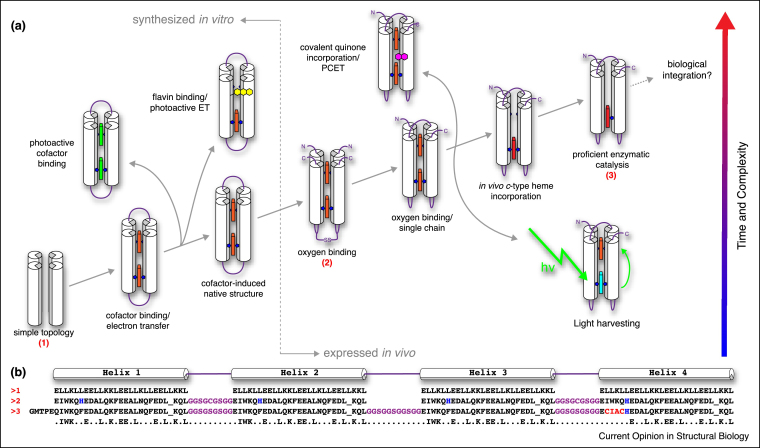
The evolution of C45 from humble beginnings. **(a)** Evolutionary diagram displaying the broad design strokes and functions of the maquettes from a simple featureless 4-helix bundle (1) [35], to the oxygen binding HP7 maquette (2) [35], to the functional *de novo* enzyme C45 (3) [22^••^]. **(b)** Sequence alignment of three maquettes from the evolutionary diagram of C45. Heme-ligating histidines are highlighted in blue, CXXC from the *c*-type cytochrome consensus motif (CXXCH) is highlighted in red, and the interhelical loops are displayed in purple.

Early maquettes were dimeric, comprising synthesized peptides each with 2 helices connected by linking loops. A subsequent design, HP7 (Figure 1(2)) has an O_2_-binding heme and features helix–loop–helix monomers linked by a covalent disulphide bond ‘candelabra’ geometry [34, 35]. More recent maquettes have utilised single polypeptide chains (Figure 1(3)) thereby avoiding the symmetry-induced constraints of earlier designs; these scaffolds have been used to reproduce oxidoreductase functions with activities comparable to their natural counterparts [22••, 36, 37]. Single-chain scaffolds have advantages including the ability to incorporate single site mutations or covalent modifications, and they can be expressed *in vivo* [36].

The maquette approach has proven effective in conferring function onto a simple scaffold, thus challenging the necessity of the complexity observed in natural proteins.

## How important is protein complexity in catalysis?

While natural proteins can be complex, *de novo* protein design has proven that we can build catalytic function onto a relatively simple scaffold. Many successful *de novo* protein designs that can perform catalysis are made up of bundles of alpha helices, with activities including the hydration of CO_2_ [6], catalase activity [38], enantiospecific hydrolysis [39^•^], oxidation and hydroxylation [40, 41], and there are some that can rescue auxotroph *E. coli* strains [39^•^]. *De novo* catalytic proteins have been assembled from more complex structures, such as the hydrolytic barrel designed by Burton and colleagues [42]. However, an advantage of the bottom-up minimalist approach taken in maquette design is that more complex structures and catalytic function can be built up iteratively. In this way it is easy to determine the function of any one amino acid, facilitating the addition of mutations. We propose that a simple helix bundle is the best starting point for many forms of catalysis, particularly as it has been demonstrated that alpha helical bundle proteins are amenable to expression *in vivo* [22^••^], whereas more complex designs may require peptide synthesis or full assembly *in vitro*.

While there are many types of catalytic activity that can be performed by *de novo* designed proteins, the remainder of this review will focus on oxidoreductase activity. Oxidoreductases are an exceptionally large and important enzyme family, with many functions involving the transfer of electrons from a donor molecule to an acceptor. Heme-containing peroxidases that catalyse substrate oxidation coupled to H_2_O_2_ reduction coordinate the catalytic heme by a single histidine side chain, leaving the 6th coordination site free to bind a substrate molecule [43]; the simplicity of this design lends itself to incorporation into a *de novo* scaffold. It is well known that the requirements for peroxidase activity are minimal. For example, the mimochrome family of artificial proteins which have been designed to maintain the properties of heme within a minimal protein scaffold [44]. They consist of two polypeptide chains <14 residues long, and heme. When the heme is 5-coordinate, mimochromes have peroxidase ability, oxidising ABTS in the presence of H_2_O_2_ [45]. Not only is such a small scaffold capable of facilitating catalytic ability, mutations to the sequence can fine tune the reactivity.

There has been much interest in incorporating these characteristics into maquettes [27, 33, 37]. *De novo* proteins containing heme C are advantageous in that the irreversible covalent binding of the heme to the protein backbone facilitates purification of the functional holoprotein after expression, and, importantly, provide an opportunity for supporting a 5-coordinate heme, with one site free for substrate binding and catalysis.

*B*-type heme-binding maquettes can be converted to a covalently-bound *c*-type by using the conserved *c*-type binding motif, CX_1_X_2_CH [36]. Despite their unnatural protein sequences, *c*-type maquettes are fully assembled in *E. coli* through the addition of a periplasmic export tag and the co-expression of the type I *c*-type cytochrome maturation (Ccm) machinery [22••, 36]. This results in the covalent binding of heme through the heme vinyl groups and protein cysteine residues [36]. The maquette C45 arose from the mutation of previous *c*-type maquette designs to produce a single-chain maquette with a mono-histidine ligated heme [22^••^]. C45 has the basic requirements of a peroxidase in that the heme cofactor is solvent exposed and can bind peroxides on its distal coordination site. Furthermore, the reaction between C45 and H_2_O_2_/ABTS (2,2′-azino-bis(3-ethylbenzothiazoline-6-sulfonic acid)) follows the kinetics of natural peroxidases, even matching the catalytic efficiency of horse radish peroxidase (HRP) operating at its optimum.

The ability to perform efficient catalysis even in an extremely simplified scaffold compared to the complexity of natural proteins leads us to pose the question: how important is a defined structure in catalysis?

## How important is a defined structure in catalysis?

Protein design has utilised the basic requirements for protein folding in which thermodynamic requirements for the assembly of secondary structure are satisfied by using simple heptad patterning of the residues comprising the alpha helices. The folding of alpha helical units into a bundle may then follow the principles of natural proteins in which the hydrophobic interior residues are hidden from the aqueous solvent [12]. Such structure is necessary over a disordered unfolded peptide sequence to ensure stability, to facilitate structure prediction and to prevent aggregation, for example for *in vivo* expression.

In nature, structure is often very important for catalysis, in which the precise arrangement of active site amino acid side chains is imperative [46, 47]. It is thought that the active site of an enzyme stabilises the transition state over the substrate, thus lowering the activation energy required for the reaction [48, 49]. There is debate as to the importance or extent of binding-induced conformational changes to align active site residue side chains for effective catalysis [50, 51]. Regardless of the precise malleability of the active site, in many cases amino acid sidechains must form precise interactions with the substrate to facilitate catalysis, and may involve acting as donors or acceptors of protons, electrons or other groups [52, 53]; electrostatic interactions are important for transition state stabilisation [54]. There are forms of catalysis in which the precise spatial arrangement of amino acid side chains at the substrate binding site is not so important, and the precise location of substrate binding is ill-defined [55]. Where catalytic cofactors are utilised, their properties must be modulated, which is often achieved through the precise alignment and the effects of nearby amino acids [56]. This has presented a challenge when it comes to the design of *de novo* enzymes, although advances in computing power for design and structure prediction are facilitating this [4^••^]. In the case of C45, despite the dynamic nature of the protein, certain characteristics imply that it is a stable, water impenetrable structure [22^••^]. Similar structural characteristics have been observed in other *de novo* [36] and natural proteins [57]. In many of these cases this may be due to the substrate conferring structural homogeneity on the active site, it remains to be experimentally determined whether this is the case for C45.

It has been proposed that conformational flexibility may have been an important mechanism in the evolution of new reactivities in early enzymes [58]. It may therefore be prudent to follow the example of nature by replicating the characteristics of early enzymes in *de novo* designs before iteration and directed evolution to refine and expand function. Additionally, *de novo* proteins could serve as models for early enzymes to gain insights into the evolution of modern enzymes.

**Figure 2:**
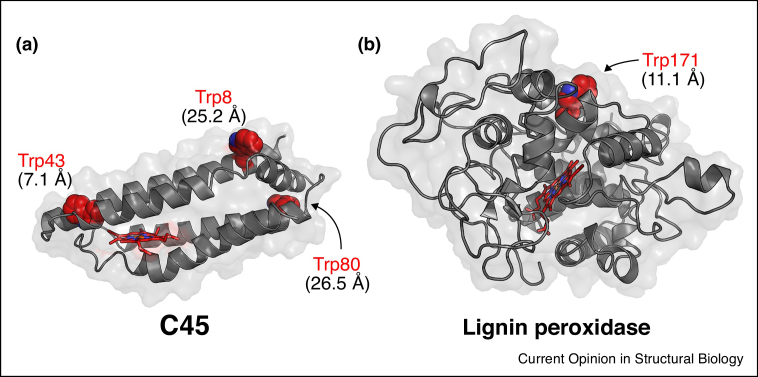
Surface tryptophan residues in both C45 **(a)** and lignin peroxidase (PDB: 1B82) **(b)** which potentially participate in long-range electron transfer from a surface-bound substrate to the protein-bound heme. Numbers in parentheses represent the edge-to-edge distances between the tryptophan side chains and the conjugated porphyrin system of the bound hemes.

**Figure 3:**
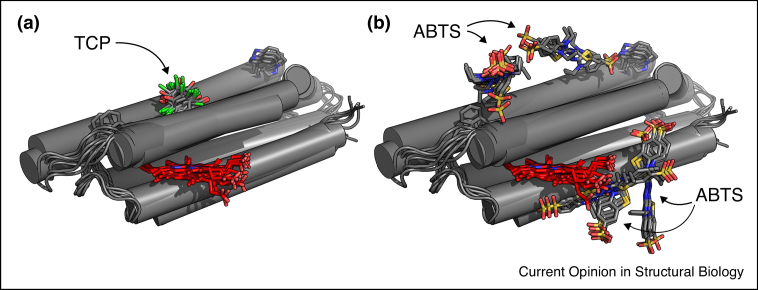
Computational analysis of potential C45-substrate binding sites. While 2,4,6-trichlorophenol (TCP) **(a)** appears to preferentially bind in one position on C45, the larger ABTS (2,2′-azino-bis(3-ethylbenzothiazoline-6-sulfonic acid)) molecule **(b)** appears to bind indiscriminately across the surface. The data presented is derived from docking analysis performed by the Bristol University Docking Engine (BUDE) [22^••^] in conjunction with molecular dynamics simulations. Overlays are shown here for representative low energy binding poses for TCP and ABTS.

Some natural peroxidases, such as ascorbate peroxidase [59], possess defined substrate-binding sites whereas others, such as lignin peroxidase, have buried hemes and do not have a well-defined cavity within the protein but instead bind the substrate on the surface [55]. In the case of surface-bound substrate, there is evidence to suggest that long-range electron transfer pathways exist to link the substrate to the buried heme *via* a catalytic surface tryptophan residue [60, 61, 62], and C45 does have surface Trp residues that could fulfil this role (Figure 2a), as does lignin peroxidase [63, 64] (Figure 2b). In the case of C45, it performs efficient catalysis without a specific binding site, and its substrate ABTS binds over the protein surface (Figure 3b). By contrast, 2,4,6-trichlorophenol (TCP), to which C45 has a lower activity, may bind in a specific place (Figure 3a). These findings indicate that it may be that binding of the heme and its immediate environment is more important for catalysis than a snug binding pocket for the substrate.

While it has been suggested that the design of recessed cavities in *de novo* enzymes is important [4^••^], the success of C45 shows that they are not always necessary for efficient catalysis. Instead it may be profitable to focus *de novo* design efforts on long-range efficient and rapid electron transfer from surface-bound substrates. Furthermore, recent research by the DeGrado group has highlighted the importance of designing the whole protein as a unit, with features far from the active site having an impact on activity [65^•^].

It is worth noting that defined structure may not be essential for *de novo* protein functions other than catalysis. The Hecht group have used a library approach of *de novo* sequences to identify proteins that can rescue auxotroph *E. coli* strains. Many of these proteins act on gene regulation [66] and, there is evidence for some of these structures that, despite being highly stable, they do not form well ordered structures *in vitro* [67].

## The advantages of substrate promiscuity in *de novo* protein design

The lack of highly specific substrate binding sites in many natural peroxidases can lead to broad substrate promiscuity, which we have observed in the case of C45, which can catalyse a variety of peroxidase substrates including guaiacol, reactive blue 4, and halogenated phenols [22^••^]. As theorised by Roy Jensen, primordial enzymes were catalytically promiscuous, then evolved to perform more specific and/or active function [68]. Thus, we may take inspiration from nature and design *de novo* enzymes without specificity, then evolve to improve or narrow selectivity. This theory of the evolution of proteins from generalists to specialists [69] has been tested using *de novo* protein design by the Hecht group [18, 70]. This was done through the use of combinatorial libraries of protein sequences designed with binary patterning principles, in which the polar and non polar nature of residues is selected and patterned to build a particular secondary structure — in this case 4-helix bundles. The majority of the proteins that were expressed could bind *b*-type heme, and most of those exhibited peroxidase activity and exhibited catalytic promiscuity. A small amount of the *de novo* proteins were hydrolases with lipase and esterase activity (activities that do not depend on bound heme). These results demonstrate that achieving catalytic ability and/or substrate binding is not difficult in unevolved protein sequences [18].

In many natural enzymes specificity is often vitally important to stringently distinguish between substrates, such is the case with restriction endonucleases [71, 72]. But for the purposes of *de novo* enzymes, this fidelity may not be strictly necessary. For example, in an industrial reaction in which only one substrate is fed into the reaction, tight control over promiscuity is not necessary as long as the desired product is produced effectively and efficiently. In fact promiscuity may be an advantage as one enzyme may be able to perform various different reactions as desired, providing the reaction energetically favours the creation of the product over the back reaction. Many natural proteins exhibit substrate promiscuity, including HRP, making it useful for many biotechnological applications [73].

While the substrate promiscuity of C45 may be advantageous, it also lends the opportunity to employ both rational protein design and directed evolution methodologies to optimize the catalytic chassis towards a selected substrate or chemical mechanism. This will be facilitated by the fact that C45 is fully assembled (and functional) *in vivo*.

## Conclusions and future directions for *de novo* enzyme design

C45 is an example of how a catalytically productive *de novo* enzyme was achieved with relative ease, without a defined substrate binding site or strict specificity. The simplicity of the C45 design provides a basic and flexible scaffold which lends itself to further modifications to achieve new functions. For an example, we anticipate that C45 may perform other natural and artificial reactions, such as carbene and nitrene transfer, and may provide a platform for accessing powerful hydroxylase chemistries.

Based on the success of the C45 design we propose that future catalytic protein design will be aided by taking a bottom-up approach, using simple scaffolds as a starting point. Rather than focussing on highly specific and efficient catalysis, we should take inspiration from the evolution of natural proteins, in which promiscuity provides the starting point for refined reactivities. While the design of active sites is a worthwhile goal, in certain cases they may not be necessary and instead treating the protein as a reactive surface with networks of interactions across the structure may be beneficial.

## References and recommended reading

Papers of particular interest, published within the period of review, have been highlighted as:

• of special interest
•• of outstanding interest
